# Periodontitis associated with plasminogen deficiency: a case report

**DOI:** 10.1186/s12903-015-0045-3

**Published:** 2015-05-14

**Authors:** Sarah H Neering, Sabine Adyani-Fard, Astrid Klocke, Stefan Rüttermann, Thomas F Flemmig, Thomas Beikler

**Affiliations:** Section of Periodontics, Heinrich-Heine University, Moorenstrasse 5, 40225 Düsseldorf, Germany; Department of Operative Dentistry Center for Dentistry and Oral Medicine (Carolinum), Goethe University Frankfurt, Theodor-Stern-Kai 7, D-60598 Frankfurt, Germany; Dean Faculty of Dentistry, Prince Philip Dental Hospital, 34 Hospital Road, Sai Ying Pun, Hong Kong; Department of Periodontics, University of Washington, 1959 NE Pacific St B307, Seattle, WA 98195 USA

**Keywords:** Periodontal disease, Periodontal therapy, Orphan disease, Plasminogen, Antibiotic therapy

## Abstract

**Background:**

Plasminogen deficiency is a rare autosomal recessive disease, which is associated with aggressive periodontitis and gingival enlargement. Previously described treatments of plasminogen deficiency associated periodontitis have shown limited success. This is the first case report indicating a successful therapy approach consisting of a non-surgical supra- and subgingival debridement in combination with an adjunctive systemic antibiotic therapy and a strict supportive periodontal regimen over an observation period of 4 years.

**Case presentation:**

The intraoral examination of a 17-year-old Turkish female with severe plasminogen deficiency revealed generalized increased pocket probing depths ranging from 6 to 9 mm, bleeding on probing over 30%, generalized tooth mobility, and gingival hyperplasia. Alveolar bone loss ranged from 30% to 50%. Clinical attachment loss corresponded to pocket probing depths. *Aggregatibacter actinomycetemcomitans, Porphyromonas gingivalis, Treponema denticola, Prevotella intermedia, Prevotella nigrescens* and *Eikenella corrodens* have been detected by realtime polymerase chain reaction. Periodontal treatment consisted of full mouth disinfection and adjunctive systemic administration of amoxicillin (500 mg tid) and metronidazole (400 mg tid). A strict supportive periodontal therapy regimen every three month in terms of supra- and subgingival debridement was rendered. The reported therapy has significantly improved periodontal health and arrested disease progression. Intraoral examination at the end of the observation period 3.5 years after non-surgical periodontal therapy showed generalized decreased pocket probing depths ranging from 1 to 6 mm, bleeding on probing lower 30%, and tooth mobility class I and II. Furthermore, microbiological analysis shows the absence of *Porphyromonas gingivalis*, *Prevotella intermedia* and *Treponema denticola* after therapy*.*

**Conclusion:**

Adjunctive antibiotic treatment may alter the oral microbiome and thus, the inflammatory response of periodontal disease associated to plasminogen deficiency and diminishes the risk of pseudomembrane formation and progressive attachment loss.

This case report indicates that patients with plasminogen deficiency may benefit from non-surgical periodontal treatment in combination with an adjunctive antibiotic therapy and a strict supportive periodontal therapy regimen.

## Background

Plasminogen (PLG) is the proenzyme of plasmin and predominately synthesized by the liver. Although the role of plasmin in intra- and extravascular fibrinolysis is well defined, it also acts as a broad spectrum proteolytic factor either by directly degrading extracellular matrix proteins, e.g. laminin, fibronection and proteoglycans, and indirectly by activating latent metalloproteinases [1,2]. Thus, it exerts crucial functions in tissue homeostasis, e.g. remodeling, angiogenesis, and wound healing [3-5]. Moreover, plasmin has also been found to play an important role in host defense against infections [1].

Plasminogen deficiency is a rare (1.6 in 1 million individuals) autosomal recessive disease caused by homozygote or compound-heterozygote mutations of the plasminogen gene PLG 6q26. There are two types of plasminogen deficiency: hypoplasminogenemia (type I PLG deficiency), in which level and activity of PLG are reduced and dysplasminogenemia (type II PLG deficiency), in which the level of immunoreactive PLG is within normal range, but the specific activity of PLG is reduced [6-9].

Clinical symptoms of type I PLG deficiency include recurrent, wood like (ligneous) pseudomembranes on mucosal surfaces of the eyes (87% of cases), upper and lower respiratory tract (33%), vagina (19%), and gastrointestinal tract (3%) [9]. In addition, approximately one third of the affected individuals suffer from pseudomembranes of the oral cavity [7,10,11].

Ligneous periodontitis is characterized by gingival enlargement and severe attachment loss, which is associated with the accumulation of amyloid-like material in the lamina propria [2]. Treatment approaches for periodontitis associated with PLG deficiency included surgical and non-surgical periodontal therapy. Periodontal surgery without previous supra- and subgingival debridement appeared initially promising, but eventually resulted in pseudomembrane regrowth. Periodontal non-surgical treatment alone i.e. without periodontal surgery or in combination with chlorhexidine mouth rinses, topical administration of plasminogen or heparin, or administration of systemic antibiotics, have been described. However, these treatments have failed to arrest periodontal disease progression in subjects with type I PLG deficiency [8,12,2].

The present case report presents the treatment of a female patient with a severe, generalized periodontitis modified by systemic factors (type I PLG deficiency) using a full-mouth disinfection approach in combination with specific adjunctive systemic antibiotic therapy aimed at altering the oral microbiome.

## Case presentation

The presented patient is a Turkish female diagnosed with type I PLG deficiency (plasminogen activity of 2%). Both of her siblings had also been diagnosed with type I PLG deficiency. At the age of 9 years, the patient presented with conjunctivitis lignosa at the Department of Pediatrics, University of Duesseldorf, where additional ligneous lesions at the mucosa of the middle ear, respiratory tract, vagina, and gingival hyperplasia were found.

**Figure 1:**
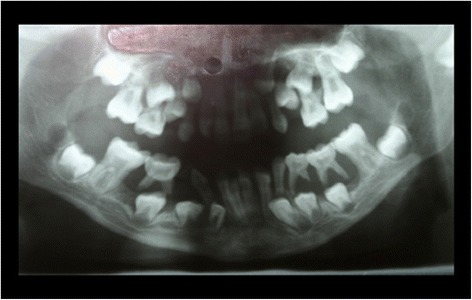
Panoramic radiograph at the age of 9 years: exhibiting signs of untreated periodontal disease.

The intraoral examination at the Department of Periodontics, University of Duesseldorf revealed erythematous and hyperplastic gingiva in the upper and lower jaw and Class 3 mobility of all of the deciduous teeth [13]. The panoramic radiograph showed severe generalized alveolar bone loss (Figure 1). The histological assessment of a gingival biopsy was taken buccaly from the second milk molar in the right lower jaw and showed a reactive squamous epithelial hyperplasia with localized fibrin precipitation and massive ulcerations. As the patient did not present for the follow-up appointments, no periodontal therapy was rendered at the time.

**Figure 2:**
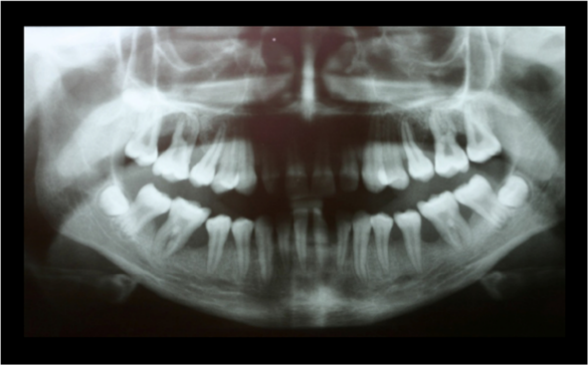
Panoramic radiograph at the age of 13 years: no periodontal therapy was rendered since the age of 9.

When she was 13 years old, the patient presented again for periodontal evaluation. At the time, the patient was receiving a systemic immunosuppressive therapy with mycophenolat-mophetil for the management of a severe pneumonia and hematocolpos. In addition, she was receiving longterm antibiotic therapy with ciprofloxacin and pyrazinomid for a pulmonary infection with multiresistant tuberculosis bacteria. The periodontal evaluation revealed signs of generalized severe periodontitis with gingival hyperplasia, ulceration and fibrinous pseudomembranes. The panoramic radiograph showed generalized horizontal bone loss of 10% to 30% at the upper and lower anterior teeth and vertical alveolar bone loss at all first molars (Figure 2). Four quadrant supra- and subgingival debridement was performed. The patient did not return for supportive periodontal therapy.

**Figure 3:**
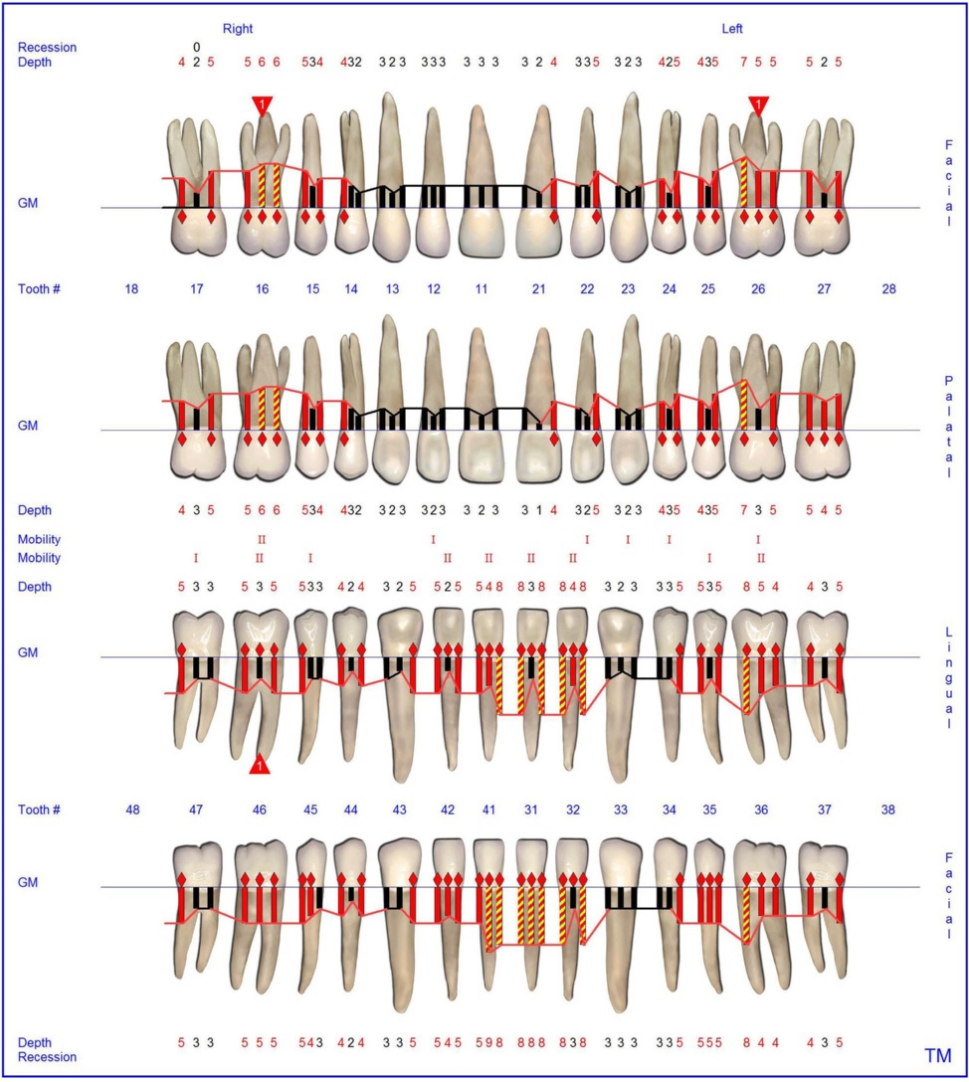
Florida Probe periodontal chart at Baseline: Periodontal measurement before full-mouth supra- and subgingival debridement in combination with an adjunctive antibiotic therapy.

**Figure 4:**
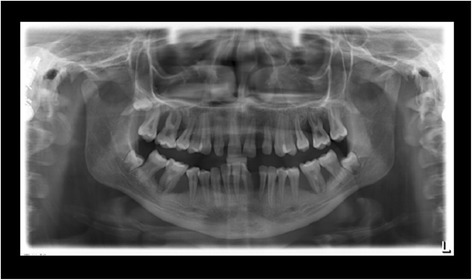
Panoramic radiograph at the age of 16 years: three years following conventional quadrantwise supra- and subgingival debridement without any supportive periodontal therapy.

**Figure 5:**
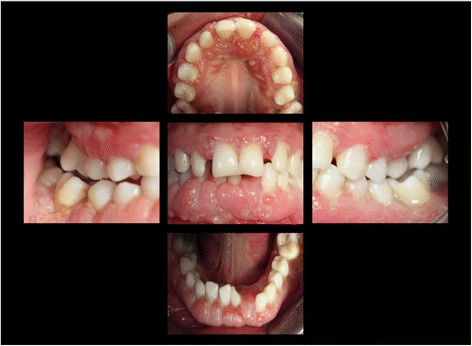
Intraoral photographs at the age of 16 years: three years following conventional quadrantwise supra- and subgingival debridement without any supportive periodontal therapy.

At the age of 16 years the patient returned. The clinical examination revealed generalized increased tooth mobility and gingival hyperplasia (Figure 3). The patient complained about severe halitosis and impaired aesthetics due to the gingival hyperplasia. Compared to the clinical and radiological assessment at 13 years of age, a progression of attachment and bone loss was noted (Figures 4 and 5). A microbiological analysis of supra- and subgingival plaque [14] revealed the intraoral presence of *Porphyromonas gingivalis, Eikenella corrodens, Prevotella intermedia, Prevotella nigrescens, Tannerella forsythensis,* and *Treponema denticola. Aggregatibacter actinomycetemcomitans* was not detected. A combined sample of supra- and subgingival plaque before non-surgical periodontal therapy was taken from the deepest periodontal pocket in each sextant and microbial species were detected by polymerase chain reaction (PCR).

**Figure 6:**
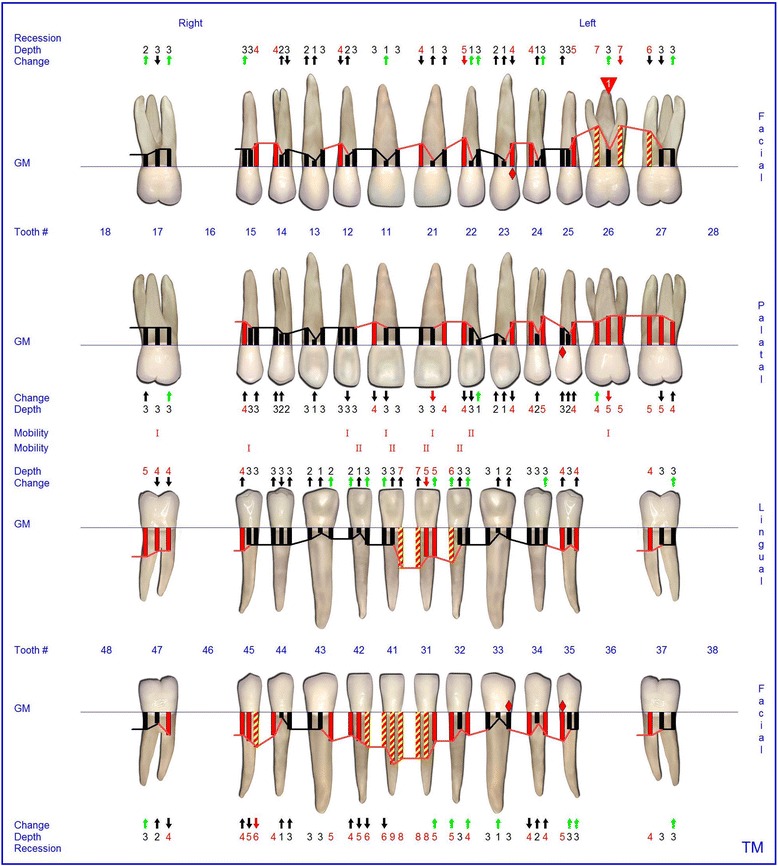
Florida Probe periodontal chart at the time of reevaluation: Six weeks after full-mouth supra- and subgingival debridement in combination with an adjunctive antibiotic therapy.

**Figure 7:**
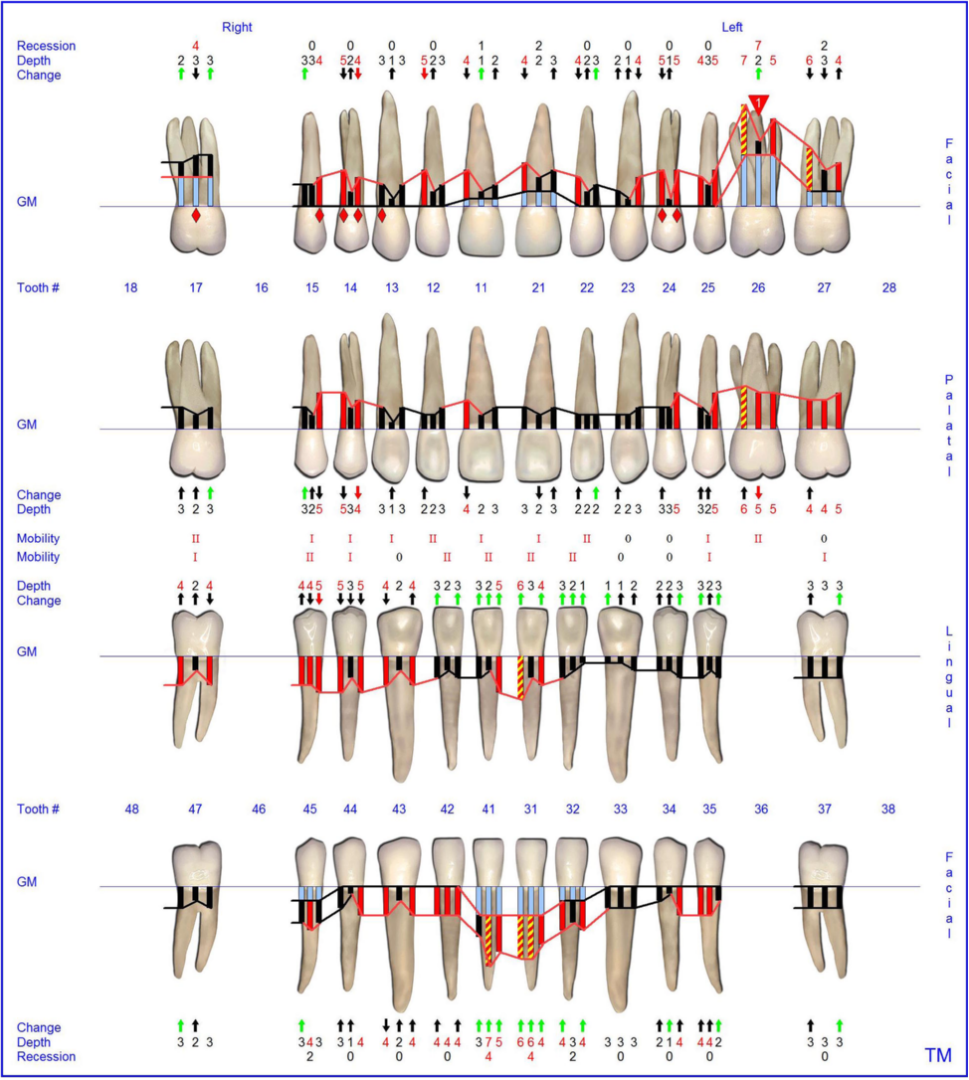
Florida Probe periodontal chart supportive periodontal therapy: 12 months following non-surgical periodontal therapy.

**Figure 8:**
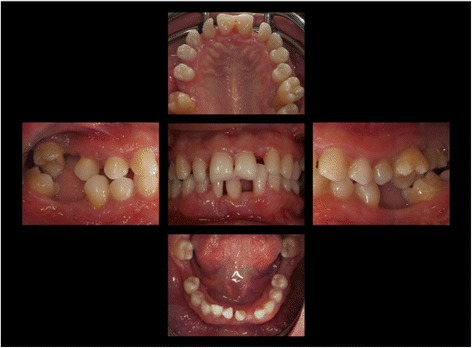
Intraoral photographs at the age of 18 years: two years following full mouth supra- and subgingival debridement in combination with an adjunctive antibiotic therapy and supportive periodontal therapy every 3 months.

**Figure 9:**
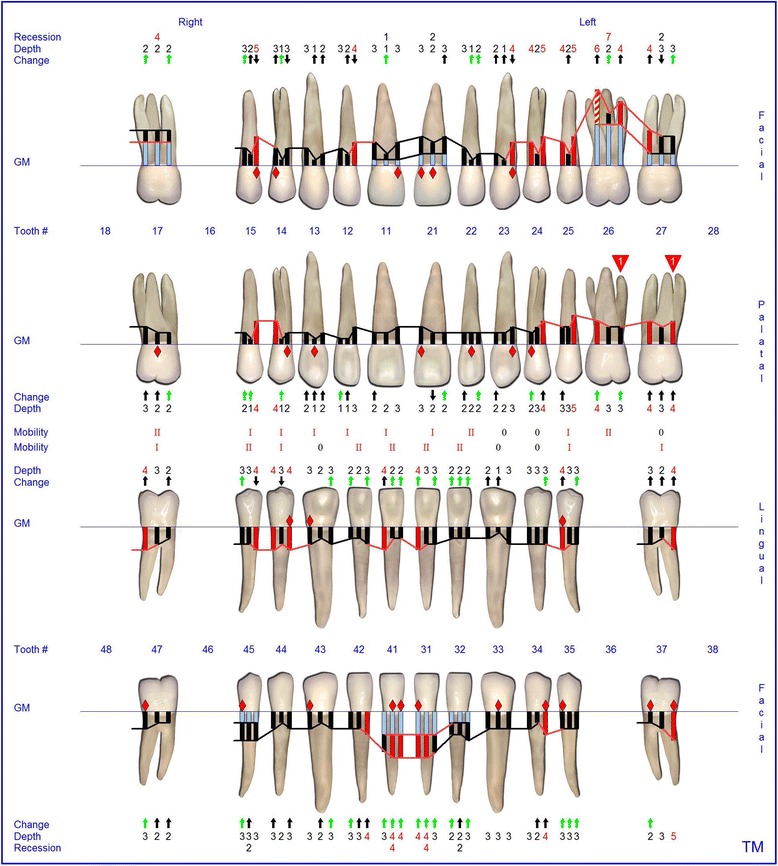
Florida Probe periodontal chart at the age of 18 years: Periodontal supportive therapy two years following full mouth supra- and subgingival debridement in combination with an adjunctive antibiotic therapy and supportive periodontal therapy every 3 months.

**Figure 10:**
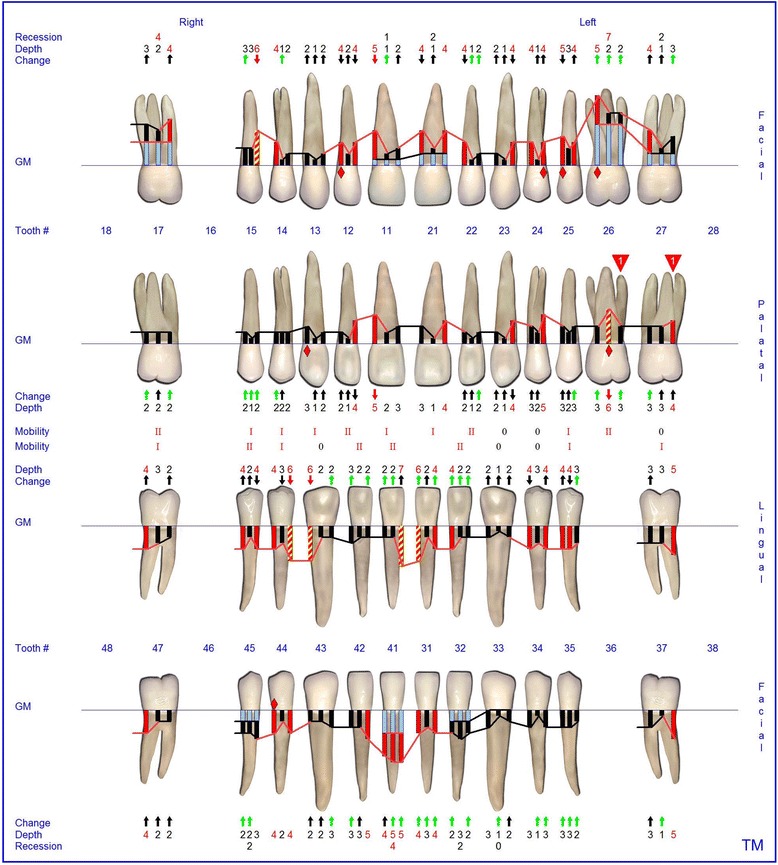
Florida Probe periodontal chart at the end of observation period: 3.5 years following full- mouth supra- and subgingival debridement in combination with an adjunctive antibiotic therapy and supportive periodontal therapy every 3 months.

**Figure 11:**
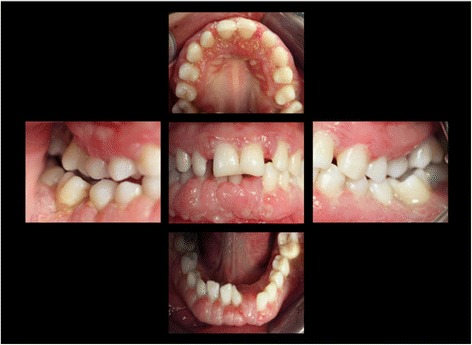
Intraoral photographs at the age of 19 years: three years following full mouth supra- and subgingival debridement in combination with an adjunctive antibiotic therapy and supportive periodontal therapy every 3 months.

**Figure 12:**
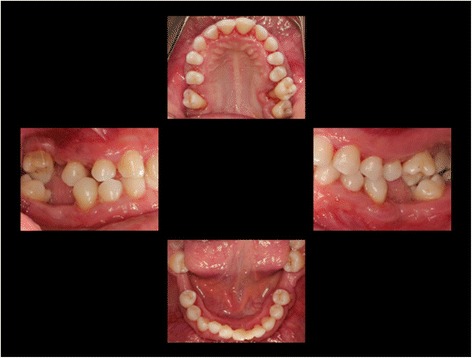
Intraoral photographs at the age of 19 years: 3.5. years following full mouth supra- and subgingival debridement in combination with an adjunctive antibiotic therapy and supportive periodontal therapy every 3 months. Situation following direct restoration with composite in the upper and lower anterior region. Note: photographs have been taken immediately following supportive periodontal therapy.

**Figure 13:**
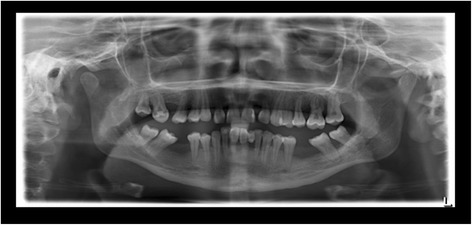
Panoramic radiograph at the age of 19 years: 3.5 years following full mouth supra- and subgingival debridement in combination with an adjunctive antibiotic therapy and supportive periodontal therapy every 3 months.

The supra- and subgingival debridement of all teeth was performed under local anesthesia within 24 hours and maxillary right and both mandibulary first molars were extracted. The patient was given oral hygiene instruction. Adjunctive antimicrobial therapy included systemic administration of amoxicillin (500 mg tid) and metronidazole (400 mg tid) and twice daily rinsing with 0.2% chlorhexidin digluconate for two weeks [15-17]. Eight weeks following the treatment the gingival hyperplasia, pocket probing depth and bleeding on probing were markedly reduced (Figure 6). The patient received supportive periodontal therapy every three months (Figure 7). At a follow-up examination at the age of 18 years, there were only minimal signs of residual gingival hyperplasia (regio 32–42) and signs of arrested periodontitis (Figures 8 and 9). A microbiological analysis showed the intraoral absence of *Aggregatibacter actinomycetemcomitans, Porphyromonas gingivalis, Prevotella intermedia* and *Treponema denticola*. Interestingly, the clinical signs of type I PLG deficiency at the ear, urogenital tract and upper respiratory tract and the eyes showed positive changes at the same time following periodontal therapy. The situation proved to be stable since (Figure 10) and at the age of 19 it was decided to improve estethics in the upper anterior region by direct restaurations with composite (Filtek Supreme XTE, 3 M Espe, Seefeld, Germany). In the lower anterior region direct composite restaurations (Filtek Supreme XTE, 3 M Espe, Seefeld, Germany) in combination with glas fiber (Ribbond THM, Ribbond, Seattle, USA) reinforced composite pontics were used (Figures 11, 12 and 13).

## Discussion

The clinical signs of ligneous periodontitis are characterized by an aggressive periodontal tissue destruction and loss of alveolar bone and teeth. The exact pathophysiology of ligneous periodontitis remains, however, unclear [18,1]. *In vitro* data and animal studies indicated that alterations in tissue repair and host defense mechanisms are responsible for the onset and the progression of periodontal destruction [1,19]. Local extracellular fibrinolysis by plasmin is required for the initial removal of the fibrin-rich matrix as well as for the remodeling of the granulation tissue and completion of wound healing [9,20,5]. Impairment of the pathway due to hypoplasminogenemia leads to fibrin accumulation and an increased inflammatory reaction. Consequently, the process of wound healing stops at the stage of granulation tissue formation and cellular proteolysis, which may then further support the invasion of pathogens. This process is notably pronounced in mucous membranes such as the periodontal tissues [12,20]. The fact that only 32% of patients who suffer from PLG type I deficiency develop ligneous periodontitis strongly supports the notion that external triggers, i.e. trauma or infection may play an additional significant role in the pathogenesis of this disease [21,10,9,22,11,2]. Therefore, the reduction of the bacterial load by an adjunctive systemic antibiotic therapy seems to be a reasonable therapy strategy to further decrease the inflammation and thus the progression of the disease. It is well known that biofilm bacteria show much greater resistance to antibiotics than their free-living counterparts. One potential reason for this increased resistance is the penetration barrier that biofilms present to antimicrobials. A complete disruption of the intraoral biofilm within a short period of time is therefore a prerequisite for the best possible antibiotic efficacy. For that reason the applied therapy approach followed the concept of full-mouth disinfection, i.e. a complete supra- and subgingival debridement within 24 hours, followed by the adjunctive systemic antibiotic therapy. In addition to that, the full mouth disinfection approach has been shown to drastically reduce periodontal pathogens in patients with generalized aggressive periodontitis [23], thus potentially further reducing the risk of disease progression. The presented treatment strategy is further supported by the guidelines of the American Academy of Periodontics as well as the German Society for Periodontology that recommend the use of a systemic adjunctive antibiotic therapy in patients with aggressive periodontitis [16,24].

Only a limited number of cases with plasminogen deficiency and oral lesions have been reported in the literature [2]. Several therapeutic approaches have been described [25-27,10,8,28] including scaling and root planning, chlorhexidine rinsing, administration of antibiotics [8,12] and periodontal surgery. These case reports, however, lack detailed information of the rendered dental therapy and the intraoral colonization with periodontal pathogens. Those very few reports about the adjunctive use of antibiotics do neither mention the type of antibiotic nor the duration of its intake, and its association to any additional periodontal treatment [29,26], thus precluding the validation of an adjunctive systemic antibiotic therapy. Most of the above mentioned reports have been described as failures due to rapid gingival regrowth and progressive bone loss [2]. Only Silva et al. report about a complete remission of the oral tissue enlargement by applying prednisolone systemically without gingivectomy [27]. However, the patient presented did not suffer from ligneous periodontitis, but from gingival enlargement only. Another case report indicated that the treatment with warfarin exerts protection against relapsing gingival hyperplasia over an observation period of 3 years in a 54 year old patient. The authors reported about a combination of gingivectomy, an administration of 20 mg doxycycline daily, and the use of a 0.12% chlorhexidine digluconate mouthrinse. One week after surgery the patient started with 5 mg warfarin daily for an indefinite time. Supportive periodontal therapy is not mentioned [18]. Thus, it is not clear, which of the rendered treatment or if their combination were responsible for the observed clinical improvement. Moreover, the described patient seemed to suffer from a clinically rather mild form of ligneous periodontitis, had lost only few teeth, and was considerably older (54 years) than other patients with ligneous periodontitis reported in the literature (average age 12–18 years). These differences may reflect variability in PLG activity due to different plasminogen gene mutations. Silva et al. [27] reported a complete regression of oral mucous lesions after systemic and topical corticosteroids. Data on the periodontal status and periodontal therapy were not published, thus hampering the evaluation of therapeutic effects on periodontal lesions.

A recent study in plasminogen-deficient mice demonstrated massive periodontal breakdown paralleled by accumulation of fibrin and neutrophils in affected periodontal tissues [1]. Interestingly, the number of colony-forming units in extracts prepared from homogenized mandibles from PLG-deficient mice was found to be approximately 100-fold higher compared with wild-type mice. The results indicate that bacterial invasion into periodontal tissues is increased in PLG-deficient mice. This finding strongly suggests that patients with PLG type I deficiency might benefit from an adjunctive systemic antibiotic therapy. Only very few case reports describe a systemic antibiotic treatment in PLG type I deficiency. However, these case reports do neither mention the type of antibiotic used nor the duration and timing of its intake [29,26] thus presenting no objective reason for the reported failure of antibiotic therapy in PLG deficient patients.

## Conclusions

In conclusion, we report on the first successful long-term clinical management of a patient with PLG deficiency type I.

This case report indicates that patients with PLG-deficiency type I may benefit from non-surgical periodontal therapy including full mouth disinfection in combination with an adjunctive antibiotic therapy and a strict supportive periodontal therapy regime every three month.

## Consent

Written informed consent was obtained from the patient for publication of this Case report and any accompanying images. A copy of the written consent is available for review by the Editor of this journal.

## Abbreviations

PLG: Plasminogen
tid: ter in die (three times a day)
PCR: Polymerase chain reaction
